# Pain trajectories in relation to incident functional limitation among older adults: A prospective cohort study

**DOI:** 10.1016/j.jnha.2025.100704

**Published:** 2025-10-07

**Authors:** Qiao Xiang, Yuxiao Li, Ziyi Zhong, Wei Huang, Jielei Chu, Taiping Lin, Birong Dong, Jirong Yue, Masoud Isanejad

**Affiliations:** a Department of Geriatrics and National Clinical Research Center for Geriatrics, West China Hospital, Sichuan University, Chengdu, 610041, Sichuan, China; b Department of Musculoskeletal and Ageing Science, University of Liverpool, Liverpool, L7 8TX, United Kingdom; c Department of Integrated Traditional Chinese and Western Medicine, Sichuan Provincial Pancreatitis Centre and West China-Liverpool Biomedical Research Centre, West China Hospital, Sichuan University, Chengdu, 610041, Sichuan, China; d School of Computing and Artificial Intelligence, Southwest Jiaotong University; e Liverpool Centre for Cardiovascular Science at University of Liverpool, Liverpool John Moores University and Liverpool Heart and Chest Hospital, Liverpool, United Kingdom

**Keywords:** Pain, Functional limitation, ADL, IADL, Group-Based trajectory modeling

## Abstract

**Objectives:**

We aimed to identify pain trajectories and examine their associations with incident functional limitation in older adults, including activities of daily living (ADL) and instrumental ADL (IADL).

**Design:**

A prospective cohort study.

**Setting:**

Community-based setting in western China.

**Participants:**

We included participants with pain score information during 2018–2022, no prevalent functional limitation by 2022, and sufficient follow-up data for functional limitation assessment during 2023–2024.

**Measurements:**

Group-based trajectory modeling was applied to identify pain trajectories based on the Numeric Rating Scale pain scores. Multivariate logistic regression models were used to assess the association between pain trajectories and incident ADL and IADL limitation. Sensitivity analyses were conducted using generalized estimating equations. Subgroup analyses were performed to assess potential interaction effects.

**Results:**

A total of 887 older adults (all aged ≥60 years) were finally included in the analytic sample, of whom 139 (15.7%) and 198 (22.3%) individuals developed incident ADL and IADL limitation during the two-year follow-up, respectively. Four pain trajectories were identified: ‘*Persistently Pain-Free*’ (43.0%), ‘*Pain Remission*’ (16.7%), ‘*Developing Mild Pain*’ (21.9%), and ‘*Persistent Mild-to-Moderate Pain*’ (18.5%). Compared to the ‘*Persistently Pain-Free*’ group, only participants in the ‘*Persistent Mild-to-Moderate Pain*’ group had a significantly higher risk of developing ADL limitation (adjusted odds ratio = 2.19, 95% CI 1.32–3.63). No significant associations were found between any pain trajectory and the risk of incident IADL limitation. No significant interactions were observed in the subgroup analyses for either ADL or IADL (P for interaction > 0.05).

**Conclusion:**

Persistent pain, even at mild-to-moderate levels, independently predicts incident limitation in ADL rather than IADL in older adults, while achieving pain remission may help prevent functional decline in ADL. These findings underscore the importance of early identification and management of persistent pain to help maintain functional independence.

## 1 Introduction

With global population aging, functional limitation has become a growing public health concern. Defined as difficulty or dependence in performing tasks essential for independent living, it is typically assessed by activities of daily living (ADL) and instrumental ADL (IADL) [1,2]. Prevalence estimates of at least one ADL or IADL limitation among older adults were reported to range from 5.1% to 44.0% or 6.0% to 40.0%, respectively [[3], [4], [5]]. Functional limitation is linked to adverse outcomes including falls, reduced quality of life, hospitalization, institutionalization, and mortality; it also places high burdens on family caregiving and healthcare utilization [[6], [7], [8], [9], [10]]. Identifying modifiable risk factors is therefore crucial for its timely and effective prevention.

Pain is a distressing sensory and emotional experience linked to actual or potential tissue damage [11]. Chronic pain is highly prevalent among older adults, affecting 25%–50% of community-dwelling individuals and up to 80% of those in institutional settings [12]. Close relationships have been established between pain and physical dysfunction, impaired mobility, or ADL/IADL disability [[13], [14], [15], [16]]. However, most research relies on single-point pain assessments, overlooking its dynamic nature. Capturing pain trajectories enables a more dynamic reflection of chronic pain, which may otherwise be mischaracterized by transient acute episodes or static measures.

The temporal course of pain can vary substantially across individuals in terms of baseline severity and the direction or magnitude of change [17,18]. Group-based trajectory modeling (GBTM), a data-driven approach, can uncover latent classes with intra-class homogeneity and inter-class heterogeneity [19,20], enabling the identification of distinct pain trajectory groups sharing similar patterns. However, most existing observational studies on pain trajectories have been conducted among clinical populations seeking healthcare for pain, potentially over-presenting more severe cases, excluding pain-free individuals at baseline, and thereby limiting the generalizability of findings to broader community populations [[21], [22], [23]]. Moreover, although pain explains disability better when treated as a heterogeneous condition rather than a single entity^18^], associations between pain trajectories and functional limitation risk remain to be clarified. Prior evidence has mostly focused on specific populations (e.g. patients with arthritis or post-surgical pain) or pain sites (e.g. low back pain), and used crude categorical measures instead of more precise continuous scales to assess pain severity [18,24,25].

To address these gaps, we aimed to characterize latent classes of pain trajectories in a community-based cohort of older adults and prospectively examine their associations with incident functional limitation, involving both ADL and IADL.

## 2 Methods

### 2.1 Study design and participants

This study was a secondary analysis of data drawn from the West China Health and Aging Trend (WCHAT) project, an ongoing longitudinal cohort study registered with the Chinese Clinical Trial Registry (ChiCTR1800018895). Community-dwelling participants aged ≥50 years were enrolled at baseline (in 2018) from multiple regions of west China according to predefined criteria, followed by annual follow-ups through on-site visits (in 2019, 2021–2024) or by telephone (in 2020) with the latest completed in 2024. On-site data collection involved questionnaires, physical examinations, and laboratory tests. Only telephone interviews were conducted in 2020 due to the COVID-19 pandemic, resulting in the unavailability of physical examination and laboratory test data in 2020. The project was approved by the Ethical Committee of Sichuan University West China Hospital and adhered to the Declaration of Helsinki. All the participants or their legal proxies provided written informed consent prior to participation. Detailed information on the cohort profile was documented elsewhere [26].

For this study, pain trajectories were constructed using pain scores collected in 2018, 2021, and 2022, with the 2022 assessment serving as the baseline for the prospective follow-up of functional outcomes from 2023 to 2024. We included participants who met the following criteria: (1) pain scores available for at least two visits among 2018, 2021, and 2022; (2) completed ADL/IADL limitation assessments in 2022; (3) had at least one follow-up assessment on ADL/IADL limitation in 2023 or 2024. Exclusion criteria were: (1) missing data for key covariates in 2018; (2) prevalent limitation in either ADL or IADL by 2022. The analytic cohort was therefore restricted to participants without baseline functional limitation.

### 2.2 Data collection

#### 2.2.1 Pain assessment

Self-reported pain was assessed through interviews beginning with the question: “Have you experienced any pain lasting for at least one month in the past six months—that is, pain that affected you most of the time during the month?” Participants who answered “no” were assigned a pain score of 0. Those who answered “yes” were asked to rate their average pain severity using the Numeric Rating Scale (NRS) ranging from 0 (no pain) to 10 (worst imaginable pain). NRS scores were conventionally categorized as 1–4 (mild), 5–6 (moderate), and 7–10 (severe) [27,28]. Pain scores from 2018, 2021, and 2022 were used for trajectory modelling.

#### 2.2.2 Functional limitation assessment

The outcome of this study was incident functional limitation in ADL or IADL separately, occurring at any follow-up in 2023 or 2024. ADL reflects basic self-care tasks essential for fundamental functioning in personal hygiene and health [1], and IADL reflects more complex tasks that allow for independent living [2]. ADL limitation was assessed using the Barthel Index including ten items (bowel control, bladder control, grooming, toileting, feeding, chair transfer, ambulation, dressing, stair climbing and bathing) [29], while IADL limitation was assessed using the Lawton IADL scale comprising eight items (food preparation, housekeeping, medication management, transportation, shopping, financial management, telephone use and laundry) [30]. Participants were asked whether they required assistance with each ADL/IADL item, and those scoring below the maximum on any item, indicating incomplete independence, were defined as having ADL/IADL limitation [31].

#### 2.2.3 Covariates

Covariates were obtained through questionnaire surveys and anthropometric measurements at baseline, encompassing demographic, socioeconomic, psychological, lifestyle, and health-related factors. These included age, sex, ethnicity, education level, occupation type, marital status, physical activity level, smoking history, alcohol consumption history, nutritional status, cognitive function, depression level, number of comorbidities, and body mass index (BMI). Data collection of the covariates is detailed in the Appendix.

### 2.3 Statistical analysis

#### 2.3.1 GBTM

Pain trajectories were identified by GBTM, a semi-parametric finite mixture modeling method, using the FlexMix package (version 2.3.20) in R [32]. To determine the optimal model specification, various functional forms for the time variable (e.g. linear, quadratic, splines) were systematically tested. The model including a quadratic function of time and baseline age, which yielded the lowest Akaike Information Criterion (AIC), was selected for subsequent trajectory modeling [33].

Models with 1–6 latent classes were tested. To improve estimation stability and reduce the risk of local maxima, each model run incorporated 25 random starting values and allowed up to 1000 iterations. The optimal number of classes was determined by a comprehensive assessment of model fit and classification quality. Model fit was evaluated using AIC, Bayesian Information Criterion (BIC), and Integrated Completed Likelihood (ICL), with lower values indicating better fit. Classification quality was assessed by entropy (with values closer to 1 indicating clearer separation) and average posterior probabilities (aiming for ≥0.7). We also considered minimum class size (aiming for ≥10% of the sample), model parsimony, practical relevance and interpretability in model selection [33,34]. Finally, each trajectory class was labeled and characterized based on its distinct pattern.

#### 2.3.2 Baseline characteristics across trajectory classes

Continuous variables were presented as mean with standard deviation (SD) for normally distributed data or median with interquartile range (Q1, Q3) for skewed distributions. Categorical variables were presented as counts and percentages (%). Differences across trajectory classes were assessed using one-way analysis of variance (ANOVA) or Kruskal–Wallis H test for normally or non-normally distributed continuous variables, and Chi-square or Fisher’s exact test for categorical variables, as appropriate. For variables with significant overall differences, post hoc pairwise comparisons were performed using Tukey’s HSD (following ANOVA) or Dunn’s test with Bonferroni correction (following Kruskal–Wallis) for continuous data, and pairwise Chi-square tests with Bonferroni correction for categorical data, where applicable.

#### 2.3.3 Associations between trajectory classes and incident functional limitation

We examined the longitudinal associations between pain trajectory classes and incident functional limitation (including ADL and IADL, respectively) using multivariate logistic regression models. To test the robustness of our findings, we conducted sensitivity analyses by repeating the primary analysis with generalized estimating equation (GEE) models, which accounted for the repeated-measures nature of the outcomes. Both logistic regression and GEE results were reported as odds ratios (ORs) with 95% confidence intervals (CIs), with four models applied. Model 1 presented unadjusted, crude estimates; Model 2 adjusted for demographic variables, including baseline age, sex (male vs. female), and ethnicity (Han vs. others); Model 3 further adjusted for socioeconomic and lifestyle factors based on Model 2, including education level (high school or above vs. middle school or lower), occupation type (agriculture/farming vs. others), marital status (married vs. widowed/divorced/single), low physical activity (yes vs. no), smoking history (yes vs. no), and alcohol consumption history (yes vs. no); Model 4 additionally controlled for health and psychological variables based on Model 3, including cognitive function (mild to severe impairment vs. normal), number of comorbidities (<2 vs. ≥2), and BMI. Subgroup analyses were conducted by age group (≤65 vs. >65 years), sex, BMI category (<24 kg/m² vs. ≥24 kg/m²), and number of comorbidities (<2 vs. ≥2) to assess potential interaction effects.

All statistical analyses were performed using R (version 4.1.3) and Python (version 3.12.8), with statistical significance defined as a two-sided P value < 0.05.

## 3 Results

**Fig. 1:**
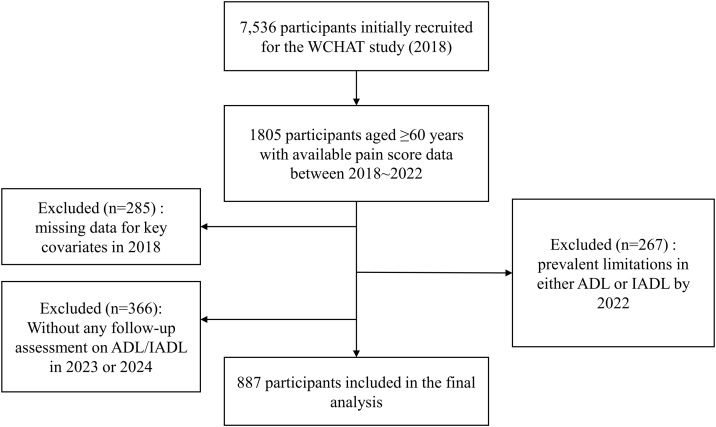
The flowchart of inclusion and exclusion of participants. Abbreviations: WCHAT, West China Health and Aging Trend; ADL, activities of daily living; IADL, instrumental ADL.

A total of 887 participants (358 males, 529 females), all aged 60 years or older (since functional limitation assessments were conducted only for older adults aged ≥ 60), were included in the final analytic sample for GBTM and subsequent analyses (Fig. 1). Compared to those excluded due to the absence of follow-up assessments for functional limitation between 2023 and 2024, the included participants were younger, more likely to be married, more physically active, and had a higher proportion of Han ethnicity at baseline, while no significant differences were observed in other lifestyle, health, or psychological factors, nor in baseline pain scores or pain severity categories (Table S1).

### 3.1 Identification of pain trajectories and baseline characteristics

**Fig. 2:**
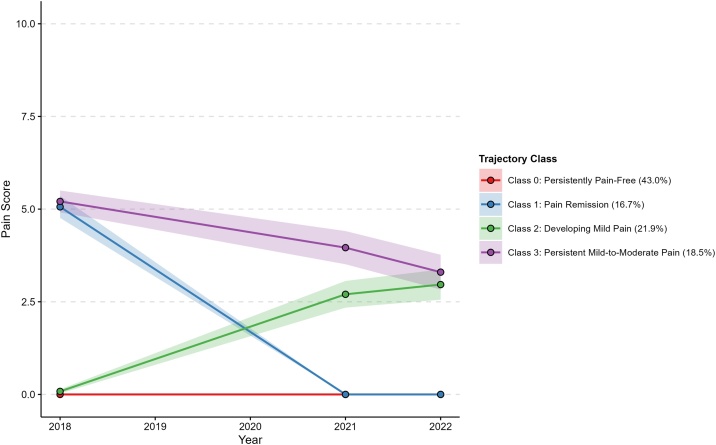
Trajectory classes of pain from 2018 to 2022.

Based on a comprehensive evaluation of model selection results (Figure S1, Table S2) and interpretability, a four-class solution for pain trajectories was ultimately selected (Fig. 2). The characteristics of the four trajectory classes (Class 0–Class 3, abbreviated as C0–C3) are summarized as follows, with the mean pain scores for each class across repeated visits presented in Table S3:

• **C0** (*Persistently Pain-Free*; n = 381 [43.0%]): Maintained a pain-free status throughout.
• **C1** (*Pain Remission*; n = 148 [16.7%]): Showed a marked decrease from moderate pain to a pain-free state, which was then sustained.
• **C2** (*Developing Mild Pain*; n = 194 [21.9%]): Experienced a gradual, slight increase from just above pain-free to approximately a pain score of 2.5, followed by a slower increase, while remaining within the mild pain range throughout.
• **C3** (*Persistent Mild-to-Moderate Pain*; n = 164 [18.5%]): Demonstrated a slight decline from just above moderate pain, with pain levels remaining within the mild-to-moderate pain range over time.

As shown in Table 1, the four trajectory classes differed significantly in their baseline distributions of sex, education level, number of comorbidities, pain scores, and pain severity categories (P < 0.05). Specifically, compared to C0, C2 and C3 had a significantly lower proportion of males and individuals with high school education or above, while C1, C2 and C3 had a higher prevalence of having ≥2 comorbidities. Baseline pain scores were the highest in C3, and were similar between C1 and C2 (see post hoc pairwise comparison results in Table S4).

**Table 1:** Baseline characteristics of participants across the pain trajectory classes.

	Class 0 (n = 381)	Class 1 (n = 148)	Class 2 (n = 194)	Class 3 (n = 164)	P value
Age	67.1 (4.9)	67.4 (5.0)	67.1 (4.7)	67.4 (5.0)	0.873
Male, n (%)	184 (48)	63 (43)	63 (32)	48 (29)	<0.001
Ethnicity, n (%)					0.164
Non-Han	173 (45)	82 (55)	90 (46)	84 (51)	
Han	208 (55)	66 (45)	104 (54)	80 (49)	
Occupation					0.159
Agriculture / Farming	274 (72)	101 (68)	153 (79)	136 (83)	
Commerce / Service	11 (3)	5 (3)	4 (2)	3 (2)	
Industry / Manufacturing	31 (8)	14 (9)	10 (5)	9 (5)	
Other	65 (17)	28 (19)	27 (14)	16 (10)	
Education: high school or above	128 (34)	39 (26)	40 (21)	27 (16)	<0.001
Marital status: married	318 (83)	124 (84)	160 (82)	133 (81)	0.904
Moderate to severe depression, n (%)	0 (0)	1 (1)	1 (1)	2 (1)	0.269
≥2 comorbidities, n (%)	84 (25)	56 (47)	73 (42)	72 (47)	<0.001
Low physical activity, n (%)	111 (35)	47 (42)	75 (44)	49 (34)	0.109
Smoking history, n (%)	82 (22)	22 (15)	36 (19)	25 (15)	0.192
Alcohol consumption history, n (%)	115 (30)	59 (40)	54 (28)	51 (31)	0.097
Mild to severe cognitive impairment, n (%)	46 (15)	10 (9)	32 (19)	25 (17)	0.132
BMI (kg/m²)	24.9 (3.2)	25.0 (3.6)	25.2 (3.5)	25.8 (3.9)	0.112
MNA-SF scores	10.9 (1.3)	11.0 (1.3)	10.8 (1.4)	10.9 (1.3)	0.642
NRS scores	0.0 (0.0)	2.0 (0.9)	1.9 (1.0)	4.3 (1.5)	<0.001
NRS category					<0.001
No pain (0)	381 (100)	0 (0)	1 (1)	0 (0)	
Mild pain (1−4)	0 (0)	146 (99)	190 (98)	101 (62)	
Moderate pain (5−6)	0 (0)	2 (1)	3 (2)	52 (32)	
Severe (7−10)	0 (0)	0 (0)	0 (0)	11 (7)	

**Note**: Data are presented as mean (standard deviation) or n (%), as appropriate. P values indicate the significance of comparisons among the classes, using one-way analysis of variance (ANOVA), Kruskal–Wallis H test, Chi-squared test, or Fisher’s exact test, as appropriate.

**Class 0**: *Persistently Pain-Free*; **Class 1**: *Pain Remission*; **Class 2**: *Developing Mild Pain*; **Class 3**: *Persistent Mild-to-Moderate Pain*.

**Abbreviations**: **BMI**, body mass index; **MNA-SF**, Mini Nutritional Assessment Short-Form; **NRS**, Numeric Rating Scale.

### 3.2 Association of pain trajectories with incident functional limitation

**Fig. 3:**
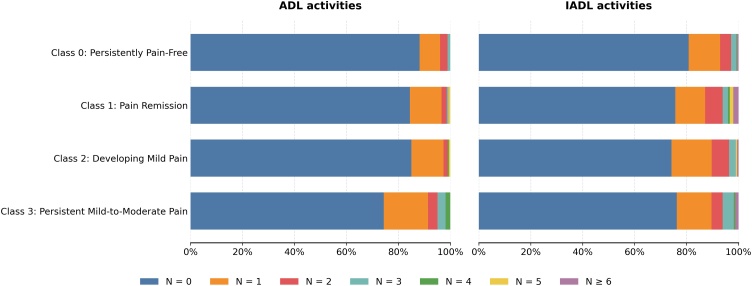
Distribution of the number of domains with incident limitation across the four pain trajectory classes for ADL and IADL. Abbreviations: ADL, instrumental activities of daily living; IADL, instrumental ADL.

During the two-year follow-up, 139 individuals (15.7%) developed incident ADL limitation (n = 45 [11.8%] in C0, n = 23 [15.5%] in C1, n = 29 [14.9%] in C2, n = 42 [25.6%] in C3), and 198 individuals (22.3%) developed IADL limitation (n = 73 [19.2%] in C0, n = 36 [24.3%] in C1, n = 50 [25.8%] in C2, n = 39 [23.8%] in C3). As shown in Fig. 3, the distribution of the number of domains with incident limitation differed significantly across the four pain trajectory classes for ADL (P = 0.003), but not for IADL (P = 0.156).

Using C0 as the reference, participants in C3 consistently showed a significantly higher risk of developing ADL limitation across Model 1–4 (in the fully adjusted Model 4: OR = 1.84, 95% CI 1.03–3.29, P = 0.040), while the relevant risks of C1 and C2 were not significantly different from that of C0 in any model. However, the risk of incident IADL limitation did not differ between C0 and any of the other trajectory classes across all models (Table 2).

**Table 2:** Logistic regression model results for associations between the pain trajectory classes and incident functional limitation, using Class 0 as the reference.

Incident Outcome	Trajectory Class	Model 1 OR (95% CI), P value	Model 2 OR (95% CI), P value	Model 3 OR (95% CI), P value	Model 4 OR (95% CI), P value
ADL	Class 1	1.37 (0.80, 2.36), 0.251	1.32 (0.76, 2.32), 0.326	1.13 (0.58, 2.21), 0.722	1.11 (0.55, 2.26), 0.763
	Class 2	1.31 (0.79, 2.17), 0.289	1.21 (0.72, 2.03), 0.475	1.15 (0.65, 2.03), 0.638	1.08 (0.59, 1.96), 0.808
	Class 3	2.57 (1.61, 4.11), <0.001	2.37 (1.46, 3.87), <0.001	2.12 (1.22, 3.67), 0.007	1.84 (1.03, 3.29), 0.040

IADL	Class 1	1.36 (0.86, 2.14), 0.188	1.39 (0.87, 2.21), 0.169	1.37 (0.79, 2.37), 0.268	1.32 (0.73, 2.36), 0.355
	Class 2	1.46 (0.97, 2.21), 0.068	1.40 (0.92, 2.13), 0.121	1.48 (0.93, 2.35), 0.099	1.39 (0.86, 2.27), 0.179
	Class 3	1.32 (0.85, 2.05), 0.222	1.24 (0.79, 1.96), 0.352	1.30 (0.79, 2.16), 0.306	1.26 (0.75, 2.14), 0.383

**Note**: Model 1 was unadjusted;

Model 2 was adjusted for baseline age, sex (male vs. female), and ethnicity (Han vs. others);

Model 3 was adjusted for education level (high school or above vs. middle school or lower), occupation type (agriculture/farming vs. others), marital status (married vs. widowed/divorced/single), low physical activity (yes vs. no), smoking history (yes vs. no), and alcohol consumption history (yes vs. no) on the basis of Model 2;

Model 4 was adjusted for cognitive function (mild to severe impairment vs. normal), number of comorbidities (<2 vs. ≥2), and BMI on the basis of Model 3.

**Class 0**: *Persistently Pain-Free*; **Class 1**: *Pain Remission*; **Class 2**: *Developing Mild Pain*; **Class 3**: *Persistent Mild-to-Moderate Pain*.

**Abbreviations**: **OR**, odds ratio; **CI**, confidence interval; **ADL,** activities of daily living; **IADL,** instrumental ADL; **BMI**, body mass index.

The sensitivity analysis using GEE yielded consistent results, showing a higher risk of incident ADL limitation in C3 than C0 (Model 4: OR = 1.71, 95% CI 1.01–2.89, P = 0.046) but no significant differences in terms of IADL (Table S5).

No significant interactions were observed in the subgroup analyses for either ADL (Figure S2) or IADL (Figure S3) (all P for interaction >0.05). Nevertheless, using C0 as the reference, the point estimates for the risk of developing ADL limitation in C3 appeared numerically higher among females, participants aged >65 years, and those with ≥2 comorbidities compared to their respective counterparts.

## 4 Discussion

In this longitudinal cohort study of community-dwelling Chinese older adults aged ≥60 years, we delineated four distinct trajectories of pain evolution. Our main finding is that older adults who experienced persistent mild-to-moderate pain had a significantly higher risk of incident ADL limitation compared to those who were consistently pain-free, while trajectories characterized by pain remission or developing mild pain did not show a significantly elevated risk. However, we did not find significant associations between pain trajectories and incident IADL limitation.

### 4.1 Characteristics of pain trajectory classes

The identified trajectories demonstrated distinct patterns of pain dynamics. The largest group (43.0%) remained consistently pain-free (C0). A substantial proportion showed notable changes over time, with 16.7% experiencing remission from moderate pain (C1), and 21.9% developing mild pain from an initially pain-free state (C2). Another 18.5% of participants endured a pattern of persistent mild-to-moderate pain (C3), despite a slight downward trend. These patterns differ from those reported in some previous studies, which were conducted in different settings, such as among institutionalized residents or patients consulting healthcare professionals, or focused on specific populations, including those with regional pain or particular diseases. Variations in age groups, sample sizes, methods and time span for pain assessment may also account for the observed heterogeneity [[21], [22], [23], [24],[35], [36], [37], [38], [39], [40]]. Notably, no trajectory characterized by severe pain was identified in our sample. This contrasts with studies conducted in clinical settings or among individuals with conditions such as knee osteoarthritis or back pain, where severe pain trajectories were more commonly observed [[21], [22], [23],^35^,^36^]. A likely explanation is that severe pain is less prevalent in general community-dwelling populations than in those who actively seek healthcare for pain-related or pathological conditions.

Baseline characteristics of the identified trajectories indicated greater vulnerability among participants experiencing pain. The classes with developing or persistent pain had higher proportions of women and individuals with lower education, and those with a history of moderate pain carried a heavier comorbidity burden. This suggests that individuals in trajectories other than persistently pain-free already faced more pronounced health and social disadvantages at baseline.

### 4.2 Persistence of pain as a key driver for ADL limitation

Compared to the reference group that remained pain-free, a significantly increased risk of incident ADL limitation was observed only in the ‘*Persistent Mild-to-Moderate Pain*’ trajectory (C3). This finding underscores the role of pain persistence as a key driver of functional decline in ADL, even if the pain is slightly alleviated but not fully resolved. This highlights the need for timely identification of individuals on this high-risk trajectory, for whom clinical management should extend beyond analgesia to include proactive ADL risk assessment and interdisciplinary interventions. These may involve multimodal pain management, early functional training, and nutritional support to prevent or delay functional decline in ADL. In contrast, the ‘*Pain Remission*’ trajectory (C1), despite beginning with moderate pain, was not associated with elevated risk, conveying a clinically encouraging message: effective pain management leading to remission may be crucial for preventing subsequent functional loss in ADL. Persistent pain often triggers a vicious cycle of activity avoidance, leading to muscle deconditioning, impaired mobility, and an increased risk of falls, all of which contribute to basic functional decline [[41], [42], [43], [44]]. Achieving pain remission may facilitate re-engagement in physical activity, thereby helping to preserve functional capacity. These findings highlight a potential window of opportunity for timely, effective pain management aimed at preventing ADL decline, shifting the goal from mere palliation to the preservation of long-term functional independence. Likewise, the ‘*Developing Mild Pain*’ trajectory (C2) was not linked to a significantly increased risk, suggesting that the transition from a pain-free state to mild pain might have not yet reached a threshold sufficient to impair ADL function within the two-year follow-up period. A Finnish study showed that individuals in intermediate-stable and high-decreasing low back pain trajectories from midlife to retirement had increased odds of ADL disability at older age [24]. Our results were generally consistent with that and provided additional evidence specific to older adults aged ≥60 years. However, our findings contrast with a study on arthritis patients where pain improvement did not correspond with improved ADL disability [18]. This discrepancy may be explained by the possibility that, in individuals with established arthritis, irreversible joint damage can weaken the relationship between pain relief and preservation of functional capacity. Although no statistically significant interactions were identified, the effect of persistent pain (C3) appeared numerically stronger among adults aged >65 years, women, and those with ≥2 comorbidities. These subgroups often have reduced functional reserve [45,46] and may therefore be more susceptible to the adverse impact of persistent pain on ADL. Future studies with larger sample sizes are warranted to determine whether age, sex, and body composition truly modify this relationship.

### 4.3 Lack of association between pain trajectories and IADL limitation

In contrast to our findings for ADL, none of the other pain trajectories were significantly associated with an increased risk of incident IADL, compared to the pain-free group. This suggests that the impact of pain trajectories on IADL function over a two-year period may be limited, possibly due to IADL’s lower sensitivity to pain or stronger dependence on other determinants. This highlights the heterogeneous effects of pain across functional domains, likely reflecting the fundamental differences between ADL and IADL regarding task complexity and functional demands.

ADL tasks rely more directly on physical capabilities such as muscle strength, balance, endurance, and joint mobility, all of which may be compromised by pain through mechanisms like activity avoidance, muscle atrophy, and fatigue. Conversely, IADL tasks depend more on higher-order cognitive functions (especially executive function and memory) and socioeconomic factors (e.g. education, occupation, and marital status) [[41], [42], [43], [44],^47^]. These were partially adjusted in our analysis and may have attenuated the effects of pain on IADL outcomes. Additionally, it is common for family members to assist with or take over IADL tasks for older adults, especially in the Chinese context. Support from spouses, relatives, and friends has been associated with lower unmet IADL needs and better IADL function [48,49], potentially masking individual’s underlying difficulties under conditions of persistent pain. Lastly, the two-year follow-up may have been insufficient to detect pain-related IADL limitation, which might emerge over a longer duration.

### 4.4 Strengths and limitations

Strengths of this study include its longitudinal design, the use of trajectory modeling to capture pain dynamics, comprehensive adjustment for potential confounders, and sensitivity analyses supporting the robustness of the findings.

Some limitations should also be noted. First, pain assessment relied on self-reported questionnaires rather than objective measures, which may introduce recall bias. However, compared to broader questions that may underestimate pain absence or simplify its intensity through a rough categorization—as discussed in previous studies [18,24,37]—our use of the NRS still offers a comparatively more refined assessment of pain burden, allowing for better reflection of diverse pain experiences with good practicality [50]. Also, our definition of pain (lasting for at least one month in the past six months) did not strictly conform to standard definitions of chronic pain (e.g., pain persisting or recurring for longer than three months according to World Health Organization) [51]. Therefore, our findings should be interpreted with caution when generalizing to chronic pain as formally defined. Second, due to the observational nature of the study, we cannot establish a definitive causal relationship between pain trajectories and functional limitation. Third, detailed data on pain characteristics (e.g. specific location, cause, frequency, duration, and treatment regimens) were not available for the current analysis, limiting further investigation in specific subgroups. However, our primary focus was on the subjective experience of overall pain, a clinically relevant and easily monitored measure in daily life. The identified trajectories may have inherently incorporated the effects of any treatments used, thus reflecting the real-world evolution of pain. Besides, older adults in China frequently use non-opioid analgesics in primary care, while strong opioid use is more restricted due to conservative prescribing practices, concerns about side effects and addiction, insurance policies, and cultural norms favoring pain endurance [52]. As evidence regarding the impact of commonly-used nonsteroidal anti-inflammatory drugs (NSAIDs) on physical or cognitive function remains limited and sometimes conflicting [53], their potential confounding effect on functional limitation may be minimal and not alter our main conclusions. Still, this gap remains to be addressed in future research. Finally, the two-year follow-up period for assessing functional outcomes is relatively short. Nevertheless, the detection of a significant association with ADL limitation within this timeframe highlights the strength and urgency of the risk posed by persistent pain, reinforcing the need for early intervention. Moreover, the short follow-up period distinguished the differential impact of pain on ADL versus IADL, suggesting that the underlying mechanisms and temporal course of functional decline may vary across domains.

In conclusion, a trajectory of persistent pain, even if mild to moderate, is an independent risk factor of incident ADL limitation but not IADL limitation in older adults. Notably, achieving pain remission may avert this risk, reinforcing the value of effective pain management. These findings underscore the importance of early identification and management of persistent pain to help maintain functional independence. Further research with larger samples, longer follow-up, and more detailed pain and treatment data is expected. In addition, exploring potential mediating pathways linking pain to functional decline may offer deeper insights into underlying mechanisms and guide more targeted interventions.

## Ethical statements

The study was approved by the Ethical Committee of Sichuan University West China Hospital and adhered to the principles of the Declaration of Helsinki. All the participants or their legal proxies provided written informed consent prior to participation.

## Declaration of Generative AI and AI-assisted technologies in the writing process

During the preparation of this work, the authors used ChatGPT in order to improve language. After using this tool, the authors reviewed and edited the content as needed and take full responsibility for the content of the publication.

## Funding statement

The study is supported by the following grants: Sichuan Province Science and Technology Innovation Base Project (2023ZYD0173); Sichuan-Chongqing Science and Technology Innovation Cooperation Plan (2024YFHZ0072); Sichuan Science and Technology Program (2024NSFSC1603); National Clinical Research Center for Geriatrics, West China Hospital, Sichuan University (Z2023LC008); China Scholarship Council Grant (#202406240227, #202406240228). The sponsors of the study had no role in study design, data acquisition, data analysis, data interpretation, or writing.

## Declaration of competing interest

The authors declare that they have no conflict of interest to this work.
