# Supplementary material for: Pain trajectories in relation to incident functional limitation among older adults: A prospective cohort study
Source: J Nutr Health Aging. 2025 Oct 7;29(12):100704. doi: 10.1016/j.jnha.2025.100704 (PMC12538691; doi:10.1016/j.jnha.2025.100704)
Supplement: Supplementary file 1 [file mmc1.docx]

## **Appendix.** Details about data collection of the covariates.

Questionnaire-based data were collected through face-to-face interviews by well-trained staffs. Physical activity was assessed using the validated China Leisure Time Physical Activity Questionnaire (CLTPAQ)^1^, a modified version of the Minnesota Leisure Time Physical Activity Questionnaire (MLTPAQ)^2^ adapted to Chinese lifestyle and culture background. As previously described, total weekly energy expenditure (kcal) was estimated based on a range of physical activities, with low activity defined as values below the sex-specific 20th percentile^3^. Nutritional status was evaluated using the Mini Nutritional Assessment Short-Form (MNA-SF), where lower scores indicate higher malnutrition risk^4^. Cognitive function was assessed via the Short Portable Mental Status Questionnaire (SPMSQ), with ≥3 errors indicating mild to severe cognitive impairment^5^. Depression was each assessed by the 15-item Geriatric Depression scale (GDS-15)^6^, with scores ≥9 indicating moderate to severe depression. Number of comorbidities was defined as the total count of chronic diseases from a predefined list, including hypertension, diabetes, coronary heart disease, stroke, chronic obstructive pulmonary disease, cancer, as well as other neurological, psychiatric, digestive, renal, musculoskeletal, and endocrine disorders. These diseases were identified via self-reported physician diagnoses from certified medical institutions in response to a structured item-by-item inquiry based on the list^7^. BMI was calculated as weight divided by height squared (kg/m^2^), using the average of two measurements for height and weight taken by trained staff.

**References**

1. Wang Y.Y., Deng C.Y., Ding D., Song Y., Lin T.P., Yue J.R., et al. [Development and validation of the China Leisure Time Physical Activity Questionnaire in the elderly]. *Practical Geriatrics*. 2019;33(03):229-233 (in Chinese).

2. Conway J.M., Irwin M.L., Ainsworth B.E. Estimating energy expenditure from the Minnesota Leisure Time Physical Activity and Tecumseh Occupational Activity questionnaires - a doubly labeled water validation. *Journal of clinical epidemiology*. Apr 2002;55(4):392-9. doi:10.1016/s0895-4356(01)00497-8

3. Ge M., Zhang Y., Zhao W., Yue J., Hou L., Xia X., et al. Prevalence and Its Associated Factors of Physical Frailty and Cognitive Impairment: Findings from the West China Health and Aging Trend Study (WCHAT). *J Nutr Health Aging*. 2020;24(5):525-533. doi:10.1007/s12603-020-1363-y

4. Kaiser M.J., Bauer J.M., Ramsch C., Uter W., Guigoz Y., Cederholm T., et al. Validation of the Mini Nutritional Assessment short-form (MNA-SF): a practical tool for identification of nutritional status. *J Nutr Health Aging*. Nov 2009;13(9):782-8. doi:10.1007/s12603-009-0214-7

5. Pfeiffer E. A short portable mental status questionnaire for the assessment of organic brain deficit in elderly patients. *Journal of the American Geriatrics Society*. Oct 1975;23(10):433-41. doi:10.1111/j.1532-5415.1975.tb00927.x

6. Almeida O.P., Almeida S.A. Short versions of the geriatric depression scale: a study of their validity for the diagnosis of a major depressive episode according to ICD-10 and DSM-IV. *Int J Geriatr Psychiatry*. Oct 1999;14(10):858-65. doi:10.1002/(sici)1099-1166(199910)14:10<858::aid-gps35>3.0.co;2-8

7. Hou L., Liu X., Zhang Y., Zhao W., Xia X., Chen X., et al. Cohort Profile: West China Health and Aging Trend (WCHAT). *J Nutr Health Aging*. 2021;25(3):302-310. doi:10.1007/s12603-020-1530-1
