# Supplementary material for: Pain trajectories in relation to incident functional limitation among older adults: A prospective cohort study
Source: J Nutr Health Aging. 2025 Oct 7;29(12):100704. doi: 10.1016/j.jnha.2025.100704 (PMC12538691; doi:10.1016/j.jnha.2025.100704)
Supplement: Supplementary file 2 [file mmc2.docx]

**Supplementary Tables and Figures**

[Table S1. Baseline characteristics between the participants excluded due to insufficient follow-up measurements and those included in the final analytical sample. 2](#_Toc208239093)

[Table S2. Summary of posterior probabilities for the pain trajectory classes. 3](#_Toc208239094)

[Table S3. The mean Numeric Rating Scale scores for each pain trajectory class across repeated visits. 4](#_Toc208239095)

[Table S4. Post hoc pairwise comparisons of baseline characteristics across the pain trajectory classes. 5](#_Toc208239096)

[Table S5. Generalized estimating equation results for associations between the pain trajectory classes and incident functional limitation, using Class 0 as the reference. 6](#_Toc208239097)

[Figure S1. Group-based trajectory modelling fit statistics for the tested number of classes (from 1 to 6). 7](#_Toc208239098)

[Figure S2. Forest plot of subgroup analyses to assess interaction effects between pain trajectories and the concerned covariates on incident ADL limitation. 9](#_Toc208239099)

[Figure S3. Forest plot of subgroup analyses to assess interaction effects between pain trajectories and the concerned covariates on incident IADL limitation. 11](#_Toc208239100)

## Table S1. Baseline characteristics between the participants excluded due to insufficient follow-up measurements and those included in the final analytical sample.

|  | **Overall (n=1253)** | **Excluded (n=366)** | **Included (n=887)** | **P value** |
| --- | --- | --- | --- | --- |
| Age | 67.7 (5.2) | 69.0 (5.6) | 67.2 (4.9) | <0.001 |
| Male, n (%) | 516 (41) | 158 (43) | 358 (40) | 0.392 |
| Ethnicity, n (%) |  |  |  | 0.003 |
| Non-Han | 640 (51) | 211 (58) | 429 (48) |  |
| Han | 613 (49) | 155 (42) | 458 (52) |  |
| Occupation |  |  |  | 0.560 |
| Agriculture / Farming | 931 (74) | 267 (73) | 664 (75) |  |
| Commerce / Service | 35 (3) | 12 (3) | 23 (3) |  |
| Industry / Manufacturing | 86 (7) | 22 (6) | 64 (7) |  |
| Other | 201 (16) | 65 (18) | 136 (15) |  |
| Education: high school or above | 322 (26) | 88 (24) | 234 (26) | 0.430 |
| Marital status: married | 1020 (81) | 285 (78) | 735 (83) | 0.047 |
| Moderate to severe depression, n (%) | 7 (1) | 3 (2) | 4 (1) | 0.159 |
| ≥2 comorbidities, n (%) | 362 (36) | 77 (33) | 285 (37) | 0.348 |
| Low physical activity, n (%) | 388 (41) | 106 (54) | 282 (38) | <0.001 |
| Smoking history, n (%) | 238 (19) | 73 (20) | 165 (19) | 0.637 |
| Alcohol consumption history, n (%) | 383 (31) | 104 (28) | 279 (31) | 0.320 |
| Mild to severe cognitive impairment, n (%) | 154 (17) | 41 (21) | 113 (15) | 0.061 |
| BMI (kg/m²) | 25.1 (3.5) | 25.0 (3.4) | 25.2 (3.5) | 0.511 |
| MNA-SF scores | 12.9 (1.3) | 12.8 (1.6) | 12.9 (1.3) | 0.917 |
| NRS scores | 1.6 (1.9) | 1.8 (2.0) | 1.5 (1.8) | 0.194 |
| NRS category |  |  |  | 0.156 |
| None (0) | 605 (48) | 168 (46) | 437 (49) |  |
| Mild (1-4) | 87 (7) | 30 (8) | 57 (6) |  |
| Moderate (5-6) | 540 (43) | 158 (43) | 382 (43) |  |
| Severe (7-10) | 21 (2) | 10 (3) | 11 (1) |  |

**Note**: Data are presented as mean (standard deviation) or n (%), as appropriate. P values indicate the significance level for comparison between the included and excluded participants, using the Student's t-test, Mann-Whitney U test, Chi-squared test or Fisher’s exact test, as appropriate.

**Abbreviations**: **BMI**, body mass index; **MNA-SF**, Mini Nutritional Assessment Short-Form; **NRS**, Numeric Rating Scale.

## Table S2. Summary of posterior probabilities for the pain trajectory classes.

| **Class** | **APP** | **Proportion of PP > 0.7 (%)** | **Proportion of PP > 0.8 (%)** | **Proportion of PP > 0.9 (%)** |
| --- | --- | --- | --- | --- |
| **0** | 0.988 | 100 | 100 | 100 |
| **1** | 0.987 | 100 | 100 | 100 |
| **2** | 0.961 | 98.5 | 92.1 | 87.3 |
| **3** | 0.982 | 98.3 | 95.7 | 93.7 |

**Note**: **Class 0**: *Persistently Pain-Free*; **Class 1**: *Pain Remission*; **Class 2**: *Developing Mild Pain*; **Class 3**: *Persistent Mild-to-Moderate Pain*.

**Abbreviations**: **APP**, average posterior probability; **PP**, posterior probability.

## Table S3. The mean Numeric Rating Scale scores for each pain trajectory class across repeated visits.

| **Class** | **Year** | **Mean ± Standard Deviation** |
| --- | --- | --- |
| Class 0 | 2018 | 0.00 ± 0.00 |
| Class 0 | 2021 | 0.00 ± 0.00 |
| Class 0 | 2022 | 0.00 ± 0.00 |
| Class 1 | 2018 | 5.06 ± 1.85 |
| Class 1 | 2021 | 0.00 ± 0.00 |
| Class 1 | 2022 | 0.00 ± 0.00 |
| Class 2 | 2018 | 0.08 ± 0.40 |
| Class 2 | 2021 | 2.70 ± 2.47 |
| Class 2 | 2022 | 2.96 ± 2.71 |
| Class 3 | 2018 | 5.21 ± 1.92 |
| Class 3 | 2021 | 3.96 ± 2.80 |
| Class 3 | 2022 | 3.30 ± 2.91 |

**Note**: **Class 0**: *Persistently Pain-Free*; **Class 1**: *Pain Remission*; **Class 2**: *Developing Mild Pain*; **Class 3**: *Persistent Mild-to-Moderate Pain*.

## Table S4. Post hoc pairwise comparisons of baseline characteristics across the pain trajectory classes.

| **Variable** | **Overall P value** | **C0 vs C1** | **C0 vs C2** | **C0 vs C3** | **C1 vs C2** | **C1 vs C3** | **C2 vs C3** |
| --- | --- | --- | --- | --- | --- | --- | --- |
| **NRS scores** | <0.001 | *** | *** | *** | ns | *** | *** |
| **Sex: male, n (%)** | <0.001 | ns | ** | *** | ns | ns | ns |
| **Education: high school or above** | <0.001 | ns | ** | *** | ns | ns | ns |
| **≥2 comorbidities, n (%)** | <0.001 | *** | *** | *** | ns | ns | ns |

**Note**: Post hoc pairwise comparisons were conducted for variables showing significant overall differences, using Tukey's HSD test, Dunn's test with Bonferroni correction, or pairwise Chi-square test with Bonferroni correction, as appropriate.

*: P< 0.05, **: P < 0.01, ***: P < 0.001, ns: not significant.

**Class 0**: *Persistently Pain-Free*; **Class 1**: *Pain Remission*; **Class 2**: *Developing Mild Pain*; **Class 3**: *Persistent Mild-to-Moderate Pain*.

**Abbreviations**: **C0-C3**; Class 0-Class 3; **NRS**, Numeric Rating Scale.

## Table S5. Generalized estimating equation results for associations between the pain trajectory classes and incident functional limitation, using Class 0 as the reference.

| **Incident Outcome** | **Trajectory Class** | **Model 1 OR (95% CI), P value** | **Model 2**  **OR (95% CI), P value** | **Model 3 OR (95% CI), P value** | **Model 4 OR (95% CI), P value** |
| --- | --- | --- | --- | --- | --- |
| **ADL limitation** | **Class 1** | 1.36 (0.80, 2.32), 0.257 | 1.28 (0.75, 2.18), 0.366 | 1.02 (0.55, 1.90), 0.954 | 1.04 (0.55, 1.94), 0.909 |
|  | **Class 2** | 1.24 (0.75, 2.03), 0.404 | 1.15 (0.69, 1.91), 0.594 | 1.02 (0.58, 1.78), 0.952 | 0.92 (0.52, 1.65), 0.789 |
|  | **Class 3** | 2.34 (1.49, 3.68), <0.001 | 2.11 (1.32, 3.35), 0.002 | 1.91 (1.13, 3.24), 0.016 | 1.71 (1.01, 2.89), 0.046 |
|  |  |  |  |  |  |
| **IADL limitation** | **Class 1** | 1.35 (0.88, 2.09), 0.172 | 1.37 (0.88, 2.12), 0.164 | 1.29 (0.76, 2.19), 0.336 | 1.25 (0.73, 2.15), 0.422 |
|  | **Class 2** | 1.37 (0.93, 2.04), 0.113 | 1.33 (0.89, 1.97), 0.164 | 1.40 (0.90, 2.18), 0.141 | 1.28 (0.80, 2.06), 0.300 |
|  | **Class 3** | 1.41 (0.92, 2.17), 0.117 | 1.34 (0.86, 2.08), 0.195 | 1.43 (0.88, 2.34), 0.149 | 1.35 (0.82, 2.21), 0.236 |

**Note**: Model 1 was unadjusted;

Model 2 was adjusted for baseline age, sex (male vs. female), and ethnicity (Han vs. others);

Model 3 was adjusted for education level (high school or above vs. middle school or lower), occupation type (agriculture/farming vs. others), marital status (married vs. widowed/divorced/single), low physical activity (yes vs. no), smoking history (yes vs. no), and alcohol consumption history (yes vs. no) on the basis of Model 2;

Model 4 was adjusted for cognitive function (mild to severe impairment vs. normal), number of comorbidities (<2 vs. ≥2), and BMI on the basis of Model 3.

**Class 0**: *Persistently Pain-Free*; **Class 1**: *Pain Remission*; **Class 2**: *Developing Mild Pain*; **Class 3**: *Persistent Mild-to-Moderate Pain*.

**Abbreviations**: **OR**, odds ratio; **CI**, confidence interval; **ADL,** activities of daily living; **IADL,** instrumental ADL**; BMI**, body mass index.


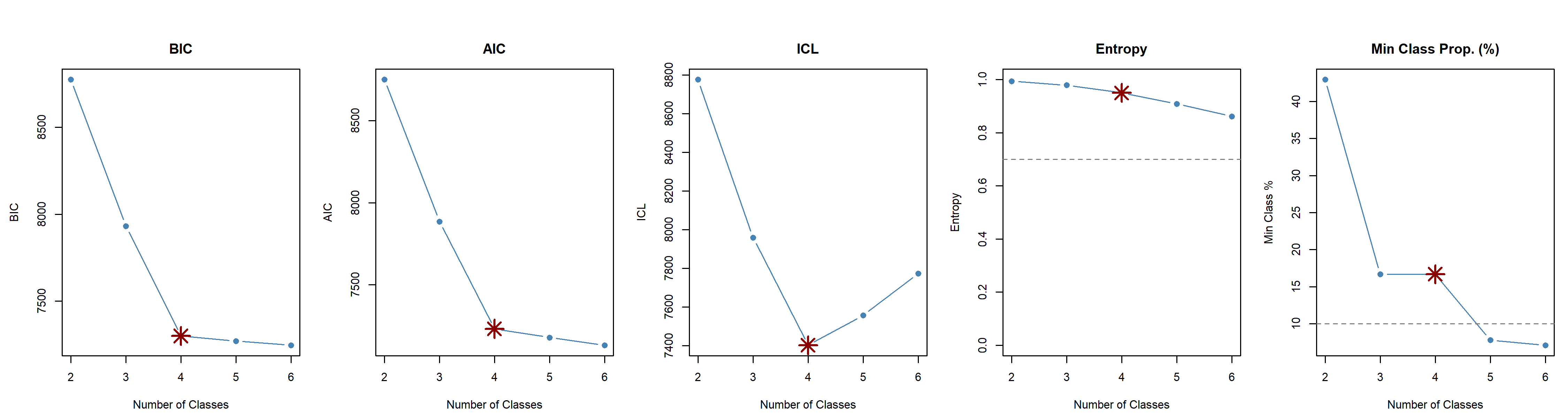


## Figure S1. Group-based trajectory modelling fit statistics for the tested number of classes (from 1 to 6).

**Abbreviations**: **BIC**, Bayesian information criteria; **AIC**, Akaike information criterion; **ICL**, Integrated Completed Likelihood; **Min Class Prop. (%)**, minimum class size.


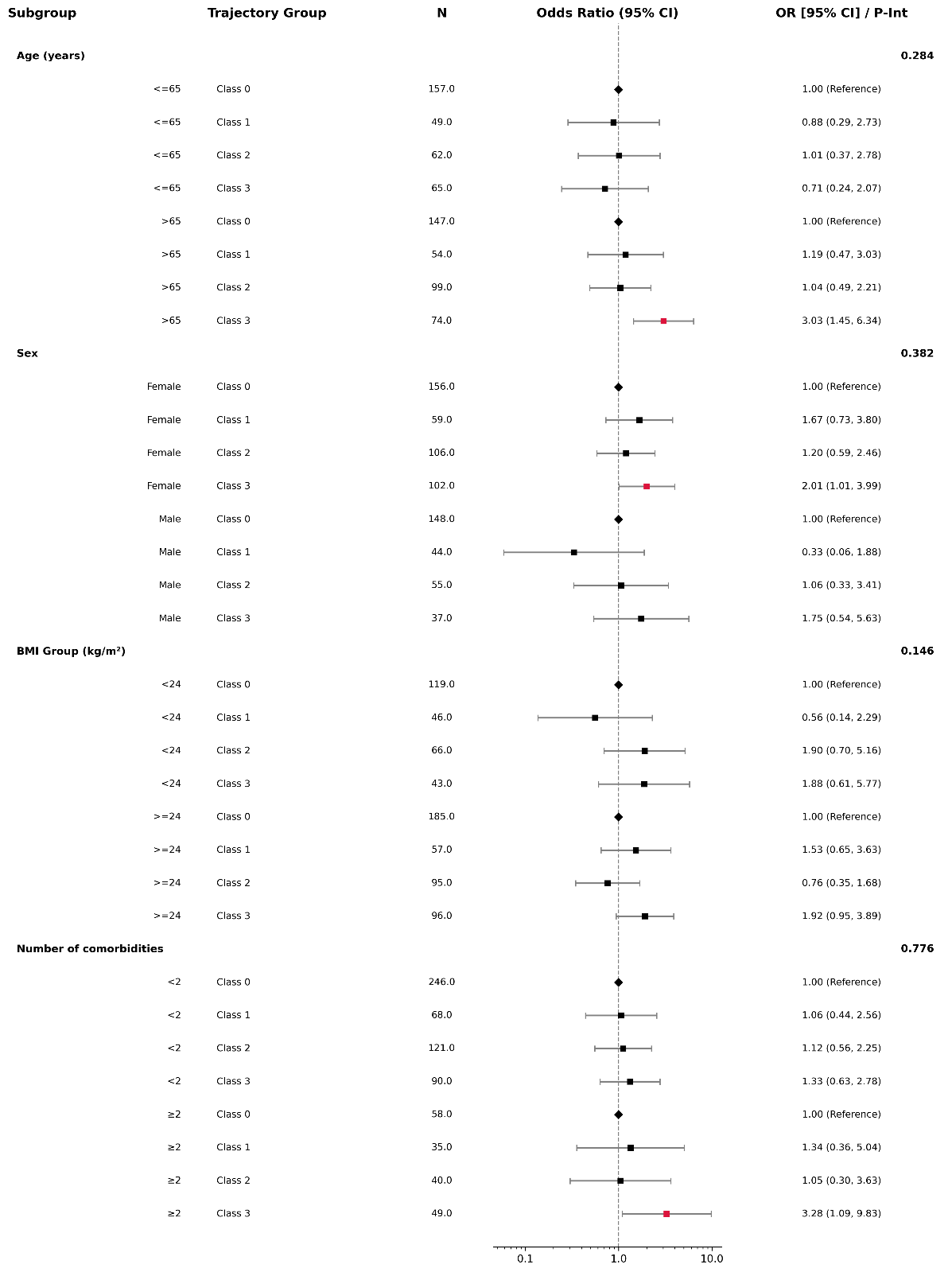


## Figure S2. Forest plot of subgroup analyses to assess interaction effects between pain trajectories and the concerned covariates on incident ADL limitation.

**Note**: Class 0 was the reference group.

**Class 0**: *Persistently Pain-Free*; **Class 1**: *Pain Remission*; **Class 2**: *Developing Mild Pain*; **Class 3**: *Persistent Mild-to-Moderate Pain*.

Estimates were based on logistic regression models, adjusting for baseline age, sex (male vs. female), ethnicity (Han vs. others), education level (high school or above vs. middle school or lower), occupation type (agriculture/farming vs. others), marital status (married vs. widowed/divorced/single), low physical activity (yes vs. no), smoking history (yes vs. no), alcohol consumption history (yes vs. no), cognitive function (mild to severe impairment vs. normal), number of comorbidities (<2 vs. ≥2), and BMI, excepting that the stratifying variable (i.e., age, sex, BMI, number of comorbidities) was excluded from the adjustment model in its respective subgroup analysis.

**Abbreviations**: **ADL**, activities of daily living; **OR**, odds ratio; **CI**, confidence interval; **P-Int**, P for interaction; **BMI**, body mass index.


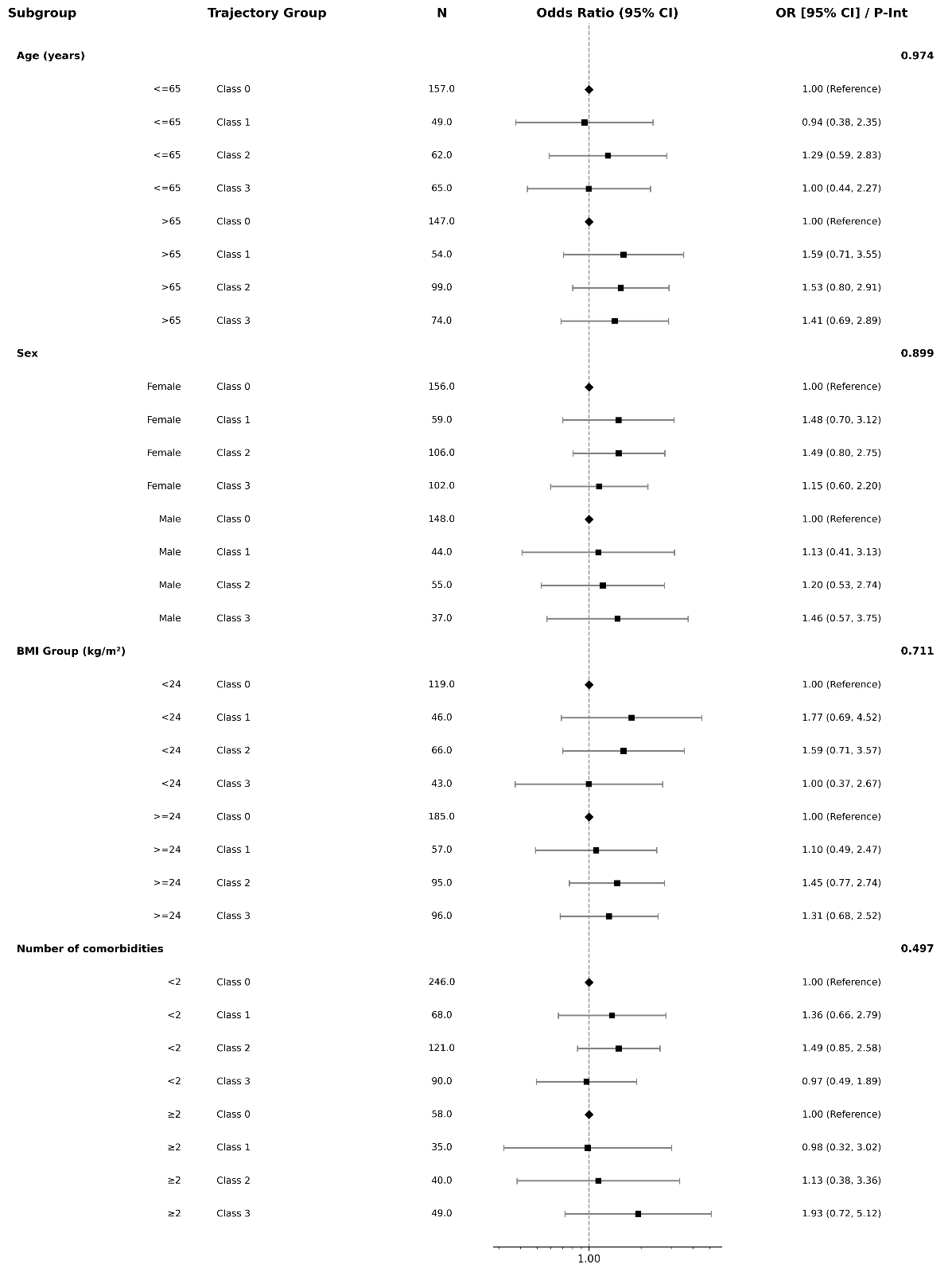


## Figure S3. Forest plot of subgroup analyses to assess interaction effects between pain trajectories and the concerned covariates on incident IADL limitation.

**Note**: Class 0 was the reference group.

**Class 0**: *Persistently Pain-Free*; **Class 1**: *Pain Remission*; **Class 2**: *Developing Mild Pain*; **Class 3**: *Persistent Mild-to-Moderate Pain*.

Estimates were based on logistic regression models, adjusting for baseline age, sex (male vs. female), ethnicity (Han vs. others), education level (high school or above vs. middle school or lower), occupation type (agriculture/farming vs. others), marital status (married vs. widowed/divorced/single), low physical activity (yes vs. no), smoking history (yes vs. no), alcohol consumption history (yes vs. no), cognitive function (mild to severe impairment vs. normal), number of comorbidities (<2 vs. ≥2), and BMI, excepting that the stratifying variable (i.e., age, sex, BMI, number of comorbidities) was excluded from the adjustment model in its respective subgroup analysis.

**Abbreviations**: **IADL**, instrumental activities of daily living; **OR**, odds ratio; **CI**, confidence interval; **P-Int**, P for interaction; **BMI**, body mass index.
